# Protective effects of mango peel extract on carboplatin/5-fluorouracil-induced parotid gland injury and hematopoietic toxicity in male Wistar albino rats: a preclinical study

**DOI:** 10.1007/s44445-025-00100-4

**Published:** 2025-12-10

**Authors:** Raneem F. Obeid, Yara Y. Mouselhy, Sally A. Abdel-halim, Mona Eltaher, Radwa T. El-sharkawy

**Affiliations:** 1 Associate Professor of Oral Biology, Faculty of Oral and Dental Medicine, Future University in Egypt, Cairo, 11835 Egypt; 2 Lecturer of Oral Pathology, Faculty of Oral and Dental Medicine, Future University in Egypt, Cairo, 11835 Egypt; 3 Chemistry of Medicinal Plants Department, National Research Center, 33 El Bohouth Street, Dokki, Giza, 12622 Egypt; 4 Assistant Professor of Applied Statistics, Faculty of Business, Ain Shams University, Cairo, Egypt; 5 Associate Professor of Oral Biology, Faculty of Dentistry, Applied Science Private University, Amman, 11937 Jordan

**Keywords:** Carboplatin, 5-fluorouracil, Mango peel, Hematopoietic toxicity, Parotid gland

## Abstract

Chemotherapeutic agents used in the treatment of head and neck cancers can induce adverse effects on healthy tissues, including hematopoietic cells. Saliva and salivary glands are also vulnerable to the toxic effects of chemotherapy. Notably, the incorporation of herbal products or their natural constituents offers the potential for mitigating the adverse effects of chemotherapy. *Mangifera indica* L., commonly known as mango, is a globally significant tropical fruit renowned for its production and consumer appeal. It possesses a range of bioactive properties, including antioxidant and anti-cancer effects. This study assessed the cytoprotective activity of mango peel extract (MPE), specifically its ability to mitigate the adverse effects of carboplatin/5-fluorouracil (5-FU) on parotid glands and hematopoietic cells. Sixty male albino rats were equally divided into control (no treatment), Drug (carboplatin/5-FU), MPE only, and Drug + MPE (carboplatin/5-FU + MPE). All treatments were administered for three weeks. Body weight, blood glucose levels, and hematological values were evaluated. Parotid gland biopsies were analyzed using H&E staining and transmission electron microscopy. The concurrent administration of MPE and carboplatin/5-FU significantly increased body weight compared to carboplatin/5-FU treatment alone. However, MPE did not counteract the decrease in blood glucose levels induced by carboplatin/5-FU. Notably, the combined use of MPE and carboplatin/5-FU resulted in a modest increase in total hematopoietic cell count compared to the substantial decrease observed with carboplatin/5-FU monotherapy. Parotid glands revealed that MPE partially restored parenchymal structure, characterized by reduced periductal fibrosis, fewer pyknotic nuclei, and milder acinar vacuolation. While some striated ducts still showed a loss of striation, excretory ducts exhibited partial improvement in cell lining in certain areas. Transmission electron microscopy confirmed these histological observations. MPE demonstrated myeloprotective effects on hematopoietic cells, improved weight loss, and reduced blood glucose levels. Furthermore, concurrent administration of MPE and carboplatin/5-FU significantly attenuated parotid gland injury induced by carboplatin/5-FU.

## Introduction

Ongoing global socioeconomic transitions are projected to cause a substantial increase in cancer incidence in the coming decades, particularly in low- and middle-income countries (Bray et al. 2012). Head and neck cancers currently represent a major global health burden (Sung et al. 2021). 5-Fluorouracil (5-FU), a common fluoropyrimidine, and Cisplatin, a foundational platinum-based alkylating agent, are cornerstones of cancer treatment (Majounie et al. 2020; Rao et al. 2022)(Zhang et al. 2022). The combination of cisplatin and 5-FU remains an established chemotherapeutic regimen for head and neck cancers (Rao et al. 2015; Wonglhow et al. 2024). Additionally, cisplatin-based concurrent chemoradiotherapy is the gold standard for treating locally advanced head and neck squamous cell carcinoma. Nevertheless, due to cisplatin’s toxicity, approximately one-third of patients are unable to complete the treatment regimen (Hanemaaijer et al. 2020).

Carboplatin, another platinum-based agent, has gained popularity as an alternative due to its improved tolerability profile. Compared to cisplatin, carboplatin exhibits greater stability and is associated with reduced nephrotoxicity, ototoxicity, gastrointestinal toxicity, neurotoxicity, and nausea(Elmorsy et al. 2024; Uehara et al. 2011). Furthermore, it offers easier administration (Dionet et al. 2002; Kua et al. 2013).

The combination of carboplatin and 5-FU thus provides an alternative regimen with a distinct toxicity profile, making it a valuable option for patients ineligible for cisplatin-based therapy (Dionet et al. 2002; Hanemaaijer et al. 2020). However, these drugs are not without adverse effects, such as myelosuppression and leukopenia (Carr et al. 2008), and other side effects, such as those involving salivary glands. The detrimental effects of 5-FU and platinum derivatives on salivary gland tissues are well documented. These chemotherapeutic drugs have been shown to induce salivary gland injury, leading to reduced saliva secretion and altered salivary protein composition, which might contribute to the occurrence and severity of oral mucositis (Bomfin et al. 2017; Fujiwara et al. 2022; Yurdabakan et al. 2022).

The efficacy of currently available therapies for preventing or treating chemotherapy-induced mucositis remains limited. Moreover, most of these agents do not target salivary gland protection or preservation of salivary gland function against the adverse effects of chemotherapy (Bensinger et al. 2008; Keefe et al. 2007; Peterson et al. 2011).

Consequently, there is an urgent need to identify novel strategies to mitigate the adverse reactions of anticancer agents and develop innovative therapies for mucositis.

Intriguingly, the integration of herbal products or their constituent natural compounds hold promise for mitigating the adverse effects of chemotherapy. Furthermore, a growing body of research underscores the resurgence of interest in plant-derived extracts within modern medicine (Galot-Linaldi et al. 2021).

Mango (*Mangifera indica* L.) is one of the most important tropical fruits worldwide in terms of production and consumer acceptance. It possesses anti-inflammatory, antioxidative, anticancer, immunostimulatory, and hepatoprotective properties (Imran et al. 2017; Shaban et al. 2023). Mango by-products, particularly mango peel, are a rich source of various health-promoting substances, such as phenolic compounds, carotenoids, and vitamin C. Additionally, mango peel exhibits a noteworthy mineral profile (Imran et al. 2017; Maldonado-Celis et al. 2019; Mercado-Mercado et al. 2018).

Therefore, this study was designed to assess the potential cytoprotective activity of mango peel extract (MPE) on parotid gland and hematopoietic cells. We aimed to determine its ability to mitigate the adverse effects of carboplatin and 5-FU in male albino rats. To the best of our knowledge, this is the first investigation into the protective effects of MPE in this context. The null hypothesis of this study is that MPE has no effect on hematopoietic cells and parotid salivary glands of rats treated with a combination of carboplatin and 5-FU.

## Material and methods

### Mango peel extract (MPE)

#### Extract preparation

Ripe peels of *Mangifera indica* L. cv. Alphonso were collected during the summer of 2024 from plants cultivated in Egypt. The plant material was identified by Dr. Kamal Zayed, Professor of Ecology, Faculty of Science, Cairo University. The collected peels were dried in an air oven at 30 °C, subsequently ground, and stored in tightly closed containers. A voucher specimen (NRC herbarium, No. 7698) was deposited for reference.

One hundred and thirty grams (130 g) of air-dried, ground ripe peels were extracted via maceration at room temperature using 70% aqueous ethanol as the solvent. A new solvent was replaced every 24 h for three consecutive times, followed by evaporation. The solvent-to-sample ratio (30:1 mL/g) and the extraction temperature (room temperature, approximately 25 °C) were used to minimize thermal degradation of phenolic and flavonoid compounds, in accordance with standard practices reported in the literature. The extraction procedure was performed in triplicate to ensure consistency and reproducibility. After complete exhaustion, the extract was filtered through Fisherbrand filter paper (QL100, 150 mm). The supernatant was then evaporated to dryness under reduced pressure at 45 °C, and the resulting extract was stored at 18 °C for subsequent total phenolic and total flavonoid analysis. To ensure compound stability, the dried extract was stored in amber-colored glass vials at 4 °C and protected from light until further analysis. These conditions are recommended to minimize the degradation of phenol and flavonoid compounds, which are sensitive to light and temperature(Munthe et al. 2023).

#### Determination of total phenolic content

The total phenolic content was determined using the Folin–Ciocalteu method (Singleton et al. 1999). Briefly, an aliquot (150 μL) of the extract solution (100 μg/mL) was added to 0.5 mL of distilled water, followed by 125 μL of Folin-Ciocalteu reagent. The mixture was vortexed and allowed to stand for 6 min, after which 1.25 mL of sodium carbonate solution (Na₂CO₃, 7% w/v) was added. The volume of the solution was adjusted to 3 mL with distilled water, thoroughly mixed, and then incubated in the dark for 90 min at ambient temperature. Subsequently, the absorbance was measured at 760 nm using a spectrophotometer. The total phenolic content was quantified using a calibration curve prepared with gallic acid (Sigma–Aldrich Chemicals Co., St. Louis, MO, USA) as the standard (concentration range: 50–250 mg/L). The results were expressed as milligrams of gallic acid equivalents per gram of dry plant weight.

#### Determination of total flavonoid content

The total flavonoid content was determined using a colorimetric assay (Tuah et al. 2017), based on the formation of a flavonoid–aluminum complex exhibiting maximum absorbance at 510 nm. Rutin (Sigma–Aldrich Chemicals Co., St. Louis, MO, USA) was used as the standard to establish a calibration curve. Briefly, 1 mL of the diluted extract was mixed with 1 mL of 2% aluminum chloride in ethanol. Following incubation at room temperature for 15 min, the absorbance was measured at 510 nm. The total phenolic content was calculated and expressed as milligrams of rutin equivalents per gram of dry plant weight.

### Experimental design

#### Animal grouping

Sixty male Wistar albino rats (180–200 gm, 6–8 weeks old) were obtained from the animal house facility of the Armed Forces Veterinary Hospital, Cairo, Egypt. Animals were housed (5/cage) in standard clean cages under controlled conditions (22–24 °C, 12 h light/12 h dark cycle) with ad libitum access to standard laboratory diet and water. All experimental procedures adhered to the guidelines established by Animal Research: Reporting In Vivo Experiments (ARRIVE). Ethical oversight was provided by the Research Ethics Committee on Living Creatures in Egypt, including the National Committee of Bioethics and the Future University Institutional Review Board (FUE.REC (31)/11–2022), which granted approval for this study.

The sample size for this study was determined using G*Power version 3.1.9.2 (Faul et al. 2007). With an effect size of 0.78, and α and β levels set at 0.05, the calculation yielded a 95% power to detect a significant difference. This resulted in an estimated sample size of 60 rats, distributed across four groups. Mortality rates were recorded for each group, with no losses observed during the trial. Additionally, the head and neck of each rat were subjected to gross examination throughout the study period (Faul et al. 2007).

The rats were randomly assigned to one of four experimental groups: a control group (receiving no treatment), a Drug group (treated solely with carboplatin and 5-FU), an MPE group (receiving MPE only), and a Drug + MPE group (receiving carboplatin and 5-FU administered simultaneously with MPE).

#### Treatment regimen

##### MPE administration

Rats received a daily oral gavage of MPE at a therapeutic dose of 100 mg/kg body weight for 21 consecutive days (El Makawy et al. 2015). To prepare the dose, MPE was dissolved in distilled water to achieve a final concentration of 100 mg/mL. the volume administrated was adjusted according to each rat’s body weight to deliver exactly 100 mg of extract/kg. Body weights were measured daily to ensure accurate dose adjustment throughout the treatment period.

##### 5-Fluorouracil (5-FU) treatment

5-FU (250 mg/5 ml) injection was obtained from Rmmpl pharma. Rats received daily intraperitoneal injections of 5-FU at a therapeutic dose of 40 mg/kg body weight for 21 days (Galot-Linaldi et al. 2021).

##### Carboplatin treatment

Carboplatin (10 mg/ml) injection was obtained from Mylan pharmaceuticals. Rats received a daily intraperitoneal injection of a therapeutic dose (100 mg/kg body weight) for 21 days (Galot-Linaldi et al. 2021).

#### Animal sacrifice

After the 21-day experimental period, all animals were euthanized by intracardiac administration of an anesthetic overdose (sodium thiopental, 80 mg/kg body weight) (Abdel Moneim et al. 2018).

### Body weight and blood glucose measurement

Body weight and blood glucose levels were measured at seven-day intervals for the 21-day study.

### Hematological evaluation

Following mild sedation with inhaled anesthesia, blood samples (1 mL) were collected from the lateral tail vein of each rat for subsequent biochemical and hematological analyses. Red blood cell count, hemoglobin concentration, white blood cell count, lymphocyte count, and platelet count were measured for each animal. Mean values for each parameter were calculated for each treatment group.

### Histological evaluation

For histopathological assessment, full-thickness biopsies of the parotid glands were taken and immediately fixed in 10% formalin for 48 h. After fixation, paraffin-embedded blocks were prepared, and sections of 5 µm thickness were obtained and stained with hematoxylin and eosin (H&E) stain according to the conventional method. For each tissue sample, 10 randomly selected, non-overlapping high-power fields (200x) were quantified per tissue block. The final score for the sample was the average score across these 10 fields.

To ensure objectivity, a blinding procedure was implemented at the sample collection stage. Tissue samples were immediately assigned a random, unique identification number by an independent third party (Technician) upon collection. This coded ID was used for all subsequent preparation (processing, embedding, and sectioning). The key linking the ID to the treatment groups was secured and remained inaccessible until all data analysis was complete.

The subsequent quantitative evaluation was also performed under blinded conditions. The pathologist responsible for all scoring was fully blinded to the treatment allocation, receiving slides labeled only with coded IDs. Blinding was maintained throughout the scoring and data entry and was only broken after the statistical analysis of the scores was finalized.

### Transmission electron microscopy (TEM)

For electron microscopy evaluation, the parotid glands were sectioned into small fragments and subjected to primary fixation in a buffered formaldehyde-glutaraldehyde solution overnight at 4 °C. Subsequently, the tissues underwent post-fixation with 1% osmium tetroxide solution in buffer for 1.5 h. Following fixation, the tissue samples were dehydrated through a graded series of ethanol solutions and embedded in a low-viscosity resin (Spurr). Semi-thin sections were then obtained and stained with toluidine blue for light microscopic examination to identify regions of interest. Subsequently, ultra-thin sections were prepared, stained with uranyl acetate and lead citrate, and examined by TEM at the Electron Microscopy Unit of Al Azhar University.

### Statistical evaluation

IBM SPSS version 27 was used for performing statistical analysis. Mean ± standard deviation was employed to express continuous variables. In addition, the Independent-Samples Kruskal–Wallis test was used for comparing means among all groups. When the Kruskal–Wallis test was significant (p < 0.05), we proceeded with post-hoc tests, specifically Pairwise Dunn’s Test for comparisons.

## Results

To comprehensively evaluate the therapeutic efficacy of MPE, we investigated its effects on key indicators of metabolic health including body weight, blood glucose levels, hematological parameters, and histopathological changes of salivary glands. Collectively, these parameters allow for a robust functional correlation between MPE’s known molecular properties and its capacity to modulate systemic and organ-level health.

### Extraction yield and quantification of Mangifera indica L. peels

The 130 g of air-dried, ground ripe peels yielded approximately 11.56 g of residue, representing 8.89% of the initial dry weight. The Total phenolic content analysis revealed that 1 mL of the 70% ethanolic extract from *Mangifera indica* L. cv. Alphonso peels contained 18.77 mg of gallic acid equivalents. The Total phenolic content analysis indicated that 1 mL of the same 70% ethanolic extract contained 0.91 mg of rutin equivalents.

### Body weight and blood glucose level results

#### Body weight

**Fig. 1:**
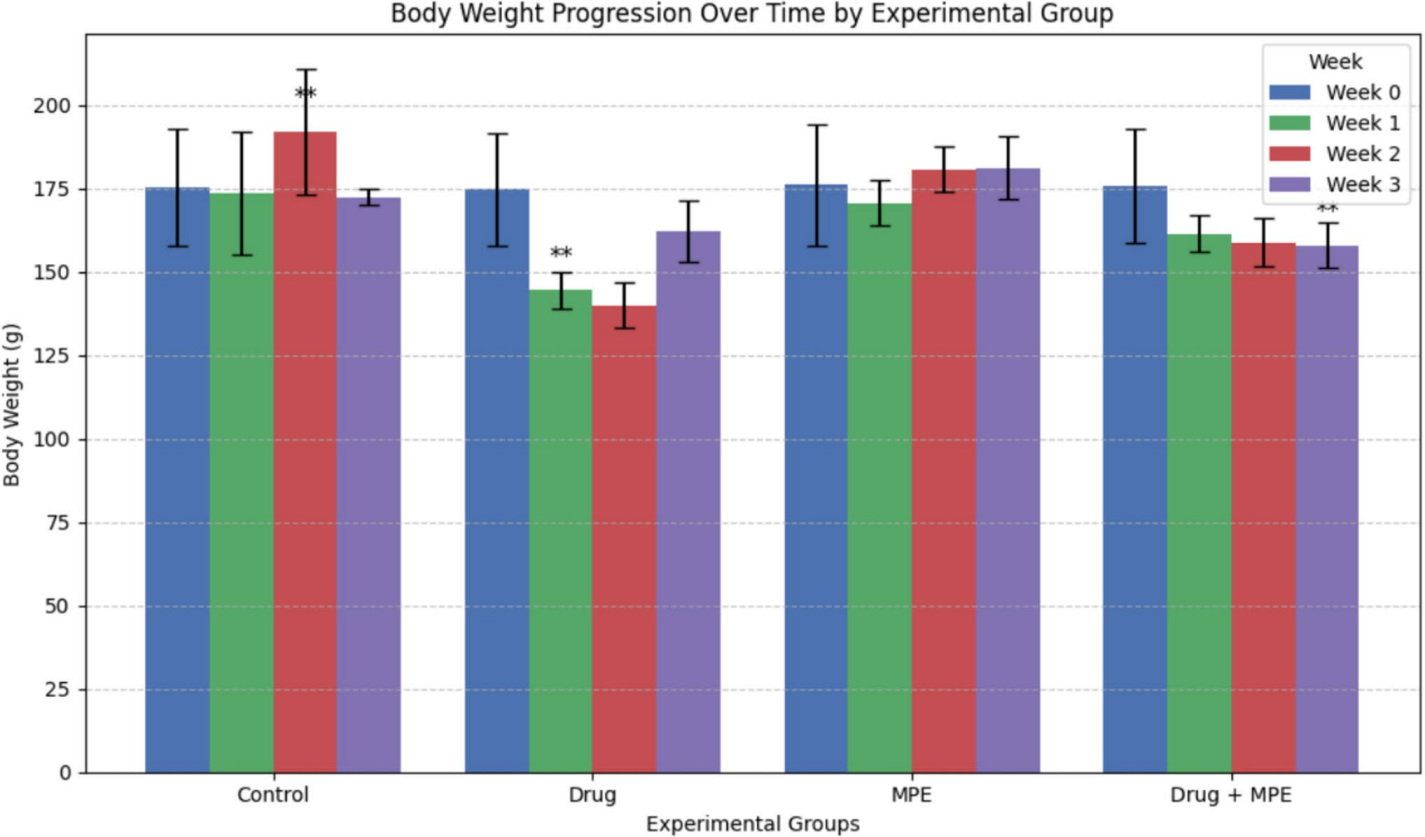
Body weight progression over time across experimental groups (n = 15 per group). Data is presented as mean ± SD. Statistical significance was assessed using Kruskal–Wallis test with Dunn’s post hoc correction. Asterisks indicate significant differences between groups: p < 0.05 (*), p < 0.001 (**)

At baseline, no significant differences in mean body weight were detected among the four experimental groups (p = 0.35). Body weight was subsequently monitored weekly, with mean body weights (g ± SD) for each group at weeks 1, 2, and 3 detailed in Table 1**, **Fig. 1**.** Notably, significant differences in body weight emerged between the Drug group and the Drug + MPE group after the first week of treatment. However, in subsequent weeks (weeks 2 and 3), while the Drug + MPE group consistently showed a numerically greater mean body weight compared to the Drug-alone group, these observed differences did not reach statistical significance. This finding indicates that concurrent administration of MPE with carboplatin/5-FU resulted in a significantly greater body weight compared to carboplatin/5-FU treatment alone, particularly evident in the initial phase of treatment.

**Table 1:** Comparative analysis of body weight across experimental groups (n = 15 per group)

Parameter	Descriptive Data/Post Hoc (Significant Groups)	Control	Drug	MPE	Drug + MPE	Kruskal–Wallis Test (Exact p-value)
Body Weight (Week 0)	Mean ± SD	174.2 ± 3.1	173.8 ± 2.9	175.1 ± 2.7	174.6 ± 2.8	—
	Range	8	7	9	8	—
Body Weight (Week 1)	Mean ± SD	173.5 ± 18.3	144.4 ± 5.42	170.6 ± 6.8	161.38 ± 5.5	0.0003**
	Range	42	16	21	16	
	Post Hoc (Significant Groups)	2	all	2	2	
Body Weight (Week 2)	Mean ± SD	191.9 ± 18.78	139.8 ± 6.8	180.7 ± 6.9	158.75 ± 7.2	0.0001**
	Range	44	18	23	17	
	Post Hoc (Significant Groups)	2,4	1,3	2,4	1,3	
Body Weight (Week 3)	Mean ± SD	172.4 ± 2.27	162.2 ± 9.13	181.1 ± 9.5	157.88 ± 6.7	0.0002**
	Range	6	28	30	16	
	Post Hoc (Significant Groups)	2,4	1,3	2,4	1,3	

* Correlation is significant at the 0.05 level (2-tailed)

Post hoc comparisons were conducted using Dunn’s test with Bonferroni correction

p < 0.05 (*), p < 0.001 (**)

#### Blood glucose level

**Fig. 2:**
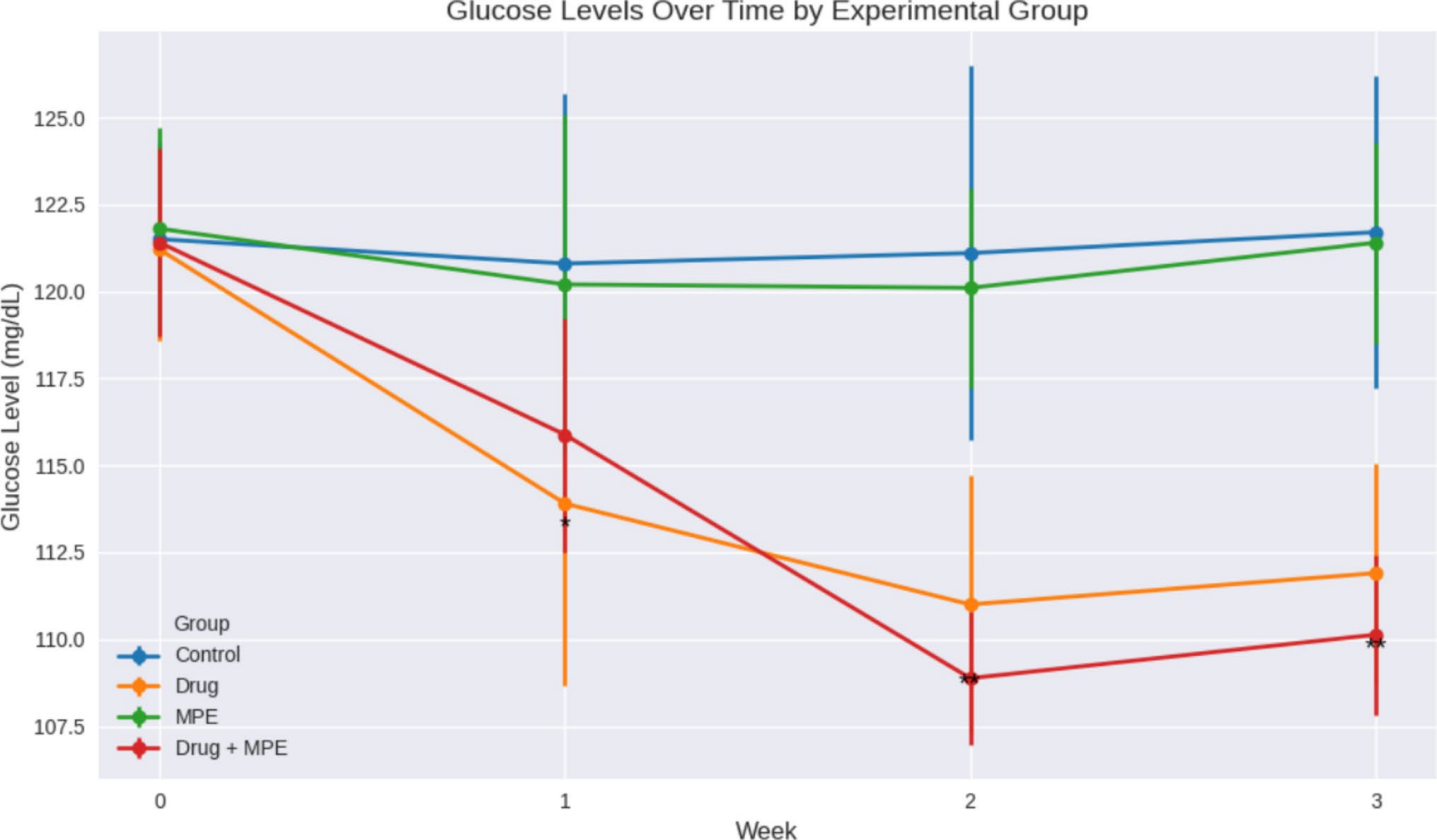
Glucose levels over time across experimental groups (n = 15 per group). Data is presented as mean ± SD. Statistical significance was assessed using Kruskal–Wallis test with Dunn’s post hoc correction. Asterisks indicate significant differences between groups: p < 0.05 (*), p < 0.001 (**)

Initially, no significant differences in blood glucose levels were detected among the four experimental groups (p = 0.11). Subsequently, blood glucose levels (mg/dL ± SD) were measured weekly **(**Table 2**, **Fig. 2**)**. Overall, we observed significant intergroup differences in blood glucose levels. However, no significant differences in blood glucose levels were observed between the Drug and Drug + MPE groups. This suggests that the co-administration of MPE with carboplatin/5-FU did not enhance the blood glucose reduction observed with carboplatin/5-FU treatment alone.

**Table 2:** Comparative analysis of glucose levels across experimental groups (n = 15 per group)

Parameter	Descriptive Data/Post Hoc (Significant Groups)	Control	Drug	MPE	Drug + MPE	Kruskal–Wallis Test (Exact p-value)
Glucose Level (week 0)	Mean ± SD	121.5 ± 2.8	121.2 ± 2.6	121.8 ± 2.9	121.4 ± 2.7	—
	Range	7	6	8	7	—
Glucose Level (Week 1)	Mean ± SD	120.8 ± 4.87	113.9 ± 5.24	120.2 ± 4.87	115.88 ± 3.36	0.012*
	Range	13	18	15	10	
	Post Hoc (Significant Groups)	2,4	1,3	2	1	
Glucose Level (Week 2)	Mean ± SD	121.1 ± 5.38	111 ± 3.7	120.1 ± 2.88	108.88 ± 1.9	0.0001**
	Range	19	12	8	5	
	Post Hoc (Significant Groups)	2,4	1,3	2,4	1,3	
Glucose Level (Week 3)	Mean ± SD	121.7 ± 4.5	111.9 ± 3.14	121.4 ± 2.9	110.13 ± 2.29	0.0002**
	Range	11	9	10	6	
	Post Hoc (Significant Groups)	2,4	1,3	2,4	1,3	

* Correlation is significant at the 0.05 level (2-tailed)

Post hoc comparisons were conducted using Dunn’s test with Bonferroni correction

p < 0.05 (*), p < 0.001 (**)

### Hematological parameters results

**Fig. 3:**
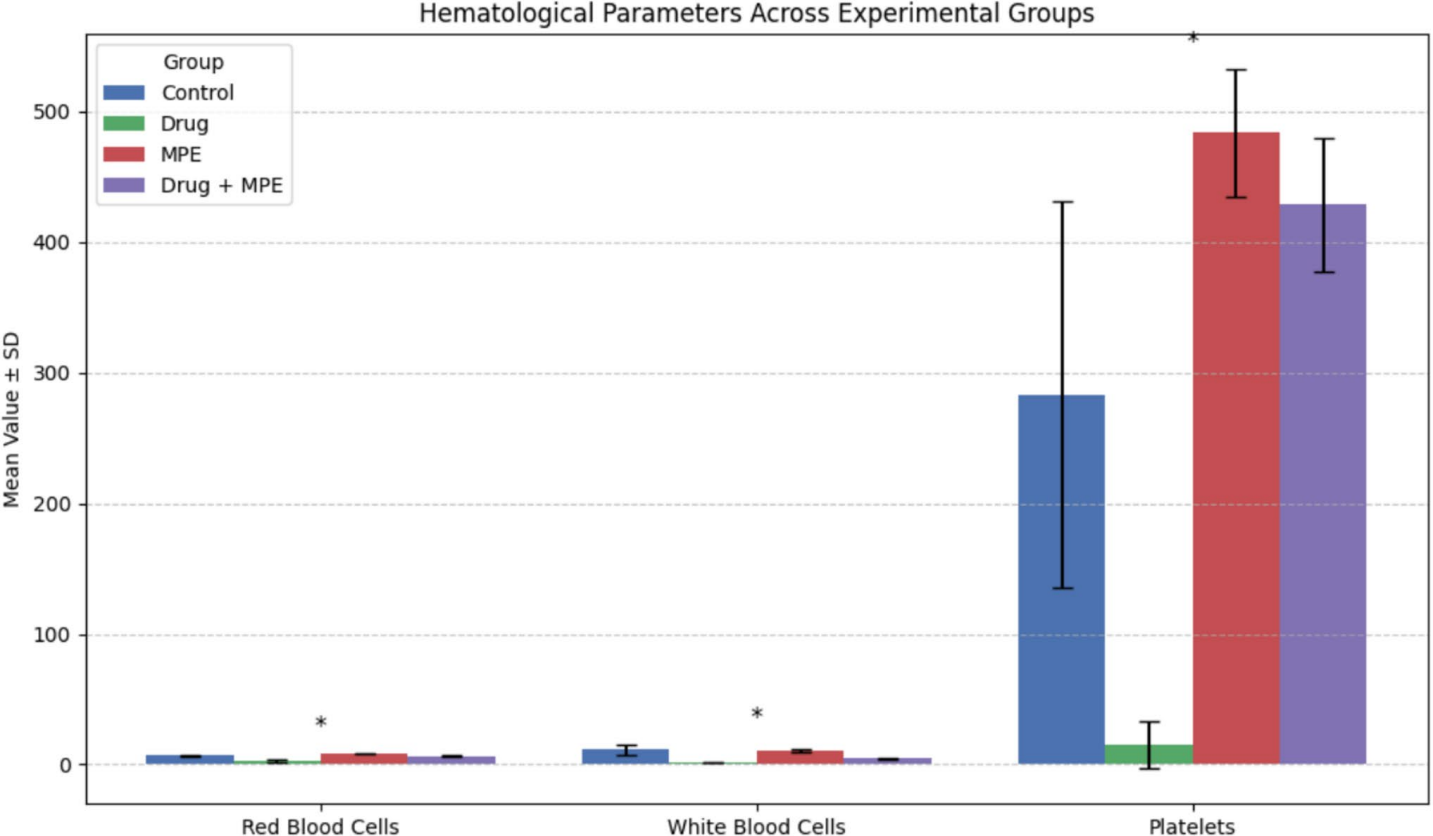
Hematological parameters across experimental groups (n = 15 per group). Data is presented as mean ± SD. Statistical significance was assessed using Kruskal–Wallis test with Dunn’s post hoc correction. Asterisks indicate significant differences between groups: p < 0.05 (*), p < 0.001 (**)

Complete blood count analysis revealed significant intergroup differences in hematological parameters (p = 0.05). The Drug group consistently exhibited markedly lower values for all assessed blood cell components compared to the other groups. In contrast, the MPE group demonstrated significantly higher mean values for red blood cell count, hemoglobin, and platelet count. The Drug + MPE group also showed a consistent numerical increase in these parameters compared to the drug-alone group, though the observed differences were statistically insignificant. **(**Table 3**, **Fig. 3**)**. These findings suggest that concurrent MPE and carboplatin/5-FU treatment resulted in a modest amelioration of the hematopoietic cell count reduction observed with carboplatin/5-FU therapy alone.

**Table 3:** Comparison of hematological parameters across experimental groups (n = 15 per group)

Parameter	Descriptive Data/Post Hoc (Significant Groups)	Control	Drug	MPE	Drug + MPE	Kruskal–Wallis Test (Exact p-value)
Red Blood Cells	Mean ± SD	6.77 ± 0.84	2.84 ± 1.6	8.4 ± 0.42	6.6 ± 0.62	0.013*
	Range	2.1	2.26	1.03	1.24	
	Post Hoc (Significant Groups)	3	3	all	3	
Hemoglobin	Mean ± SD	12.1 ± 1.8	4.5 ± 2.5	14.3 ± 0.35	12.7 ± 1.05	0.009*
	Range	3.3	3.6	0.8	2.1	
	Post Hoc (Significant Groups)	3	3	all	3	
White Blood Cells	Mean ± SD	11.34 ± 3.66	1.41 ± 0.2	10.9 ± 1.05	4.5 ± 0.65	0.021*
	Range	8.54	0.28	2.64	1.21	
	Post Hoc (Significant Groups)	2,4	1,3	2,4	1,3	
Lymphocytes	Mean ± SD	9.3 ± 3.8	1.18 ± 0.17	9.18 ± 0.88	3.2 ± 0.18	0.020*
	Range	8.9	0.24	2.07	0.36	
	Post Hoc (Significant Groups)	2,4	1,3	2,4	1,3	
Platelets	Mean ± SD	283.2 ± 147.7	15 ± 18.38	483.4 ± 48.7	428.3 ± 51.16	0.018*
	Range	333	26	115	102	
	Post Hoc (Significant Groups)	3	3	1,2	—	

* Correlation is significant at the 0.05 level (2-tailed)

Post hoc comparisons were conducted using Dunn’s test with Bonferroni correction

p < 0.05 (*), p < 0.001 (**)

### Histological results

**Fig. 4:**
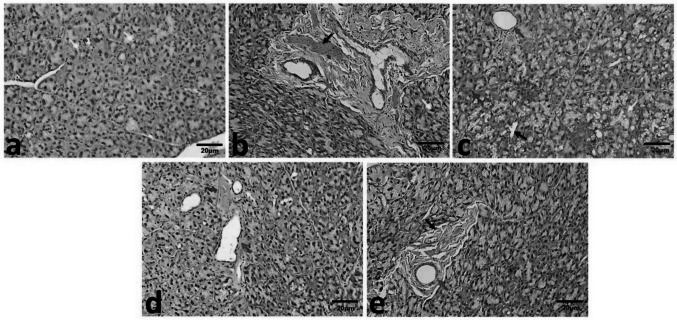
Photomicrograph of the parotid salivary gland revealing in Control group, intact parotid parenchyma with connective tissue septa and serous acini with rounded, basally located nuclei (yellow arrow) (**a**). In Drug group, structural alterations were observed, including pyknotic nuclei in acinar cells (yellow arrow) (**b**), acinar vacuolation (yellow arrow) (**c**), coalescence of acini with loss of acinar outline (blue arrow) (**c**), marked periductal fibrosis (green arrow) (**b**), loss of striation and lumen widening (red arrow), and discontinuity in the lining of striated ducts (black arrow) (**c**), disrupted lining of the excretory duct with stagnated secretion (red arrow) (**b**), and dilated blood vessels (black arrow) (**b**). Drug + MPE group showed partial restoration of the parenchymal structure, except in some areas that exhibited pyknotic nuclei in acinar cells (green arrow) (**d**) and mild acinar vacuolations (green arrow) (**e**), loss of striation in striated ducts in some areas (yellow arrow) (**d**), the excretory duct displayed flattened lining in some areas (red arrow) (**d**), and improved lining in other areas (red arrow) (**e**), vascular congestion (blue arrow) (**d**), and less marked periductal fibrosis (black arrow) (**e**) (H&E Original magnification × 200)

Histological examination of H&E sections from Control and MPE groups revealed intact parotid parenchyma with connective tissue septa. The parenchyma in both groups exhibited serous acini lined by pyramidal cells with basal nuclei **(**Fig. 4a**)**.

Conversely, the Drug group exhibited structural alterations and damage in the parotid tissue. These included dilated blood vessels and extravasated red blood cells **(**Fig. 4b**)**, as well as coalesced acini with loss of acinar outline **(**Fig. 4c**)**, displaced pyknotic nuclei **(**Fig. 4b**)**, marked acinar vacuolation **(**Fig. 4c**)**, and periductal fibrosis **(**Fig. 4b**)**. Furthermore, the glandular ducts displayed considerable alterations. Striated ducts showed loss of basal striation, flattened nuclei, perinuclear vacuolation, and discontinuity of lining **(**Fig. 4c**)**. Excretory ducts were similarly affected, exhibiting a disrupted lining with loss of pseudostratification, flattened nuclei, stagnant secretions, and inflammatory cellular infiltration **(**Fig. 4b**)**.

In contrast to the Drug group, the Drug + MPE group displayed partial restoration of the parenchymal structure. While some areas still showed vascular congestion **(**Fig. 4d**)**, we observed less marked periductal fibrosis **(**Fig. 4e**)**, fewer pyknotic nuclei, and only mild acinar vacuolation **(**Fig. 4d**)**. Regarding the ducts, striated ducts in the Drug + MPE group exhibited a loss of striation in certain areas **(**Fig. 4d**)**. Meanwhile, excretory ducts showed partial improvement of their cell lining in some regions **(**Fig. 4e**)**, though they remained unchanged in others **(**Fig. 4d**)**.

### Transmission electron microscope results

**Fig. 5:**
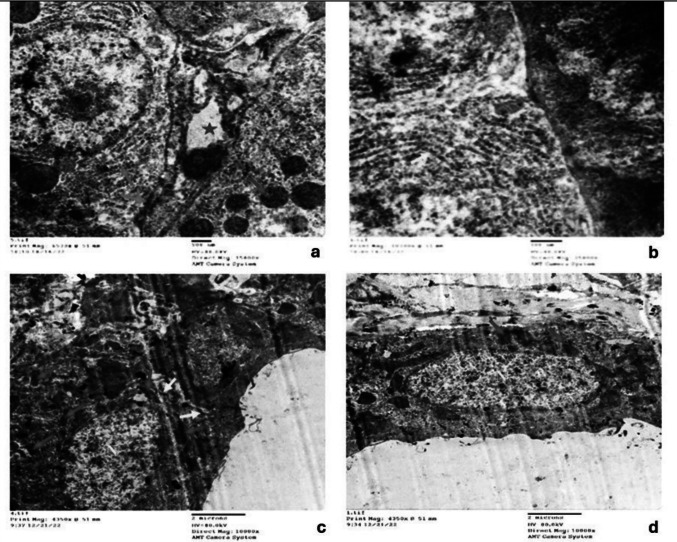
A photomicrograph of parotid salivary gland in Control group showing: myoepithelial cells along the acinar cells (*) attached with desmosome (blue arrow), secretory zymogen granules (red arrow) and well-organized RER cisternae (green arrow) (**a)** (Original mag. × 15,000), desmosomal basal attachment (red arrows) with basal folding stacked with RER (yellow arrow) in acinar cell (**b)** (Original mag. × 25,000). Open-faced nuclei (N) **(c)**, basally located mitochondria (red arrows) (**c**), and interdigitation between adjacent cells with desmosome in striated duct cells (yellow arrows) (**c**) (Original mag. × 10,000). Excretory duct demonstrating open-faced nuclei (N), mitochondria (red arrow) (**d**) (Original mag. × 10,000)

Ultrastructural examination of Control and MPE groups revealed similar features in the acinar cells. These cells exhibited a pyramidal shape with basal spheroidal nuclei, parallel arrays of rough endoplasmic reticulum (RER), oval mitochondria, and a Golgi apparatus (Fig. 5a). Membrane-bound secretory zymogen granules of varying sizes and electron densities occupied the cytoplasm (Fig. 5a). Additionally, the acinar cells displayed basal attachment via desmosomes and basal folding stacked with RER (Fig. 5b). Myoepithelial cells were observed along the acinar cells, characterized by an open-faced nucleus and attachment by desmosomes (Fig. 5a). The striated duct cells exhibited an open-faced nucleus and basally located mitochondria, with interdigitating between adjacent cells displaying desmosomal attachment (Fig. 5c). Excretory duct cells demonstrated open-faced nuclei and normal cristae of mitochondria (Fig. 5d).

**Fig. 6:**
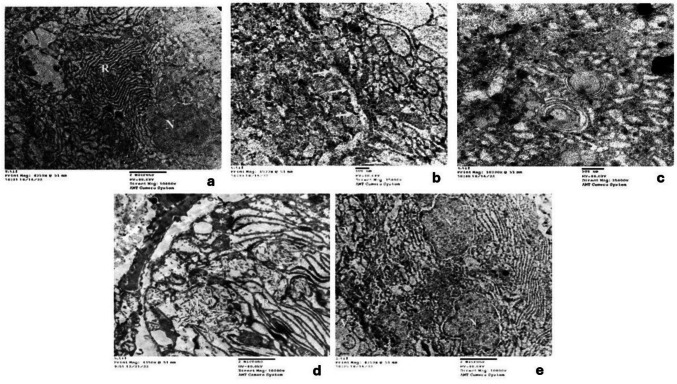
A photomicrograph of parotid salivary gland in Drug group showing: dilated RER, detachment of ribosomes (R) (**a**), cytoplasmic vacuolations (Blue arrow) (**a**), clumping of chromatin and irregular nuclear membrane (N) swollen mitochondria (Red arrow) (**a**) (Original magX 10000), loss of attachment between acinar cells (yellow arrows), dilated RER, detachment of ribosomes (R) (red arrow) (**b**) (Original magX 15000) and enlarged whorled mitochondria in acinar cell (red arrows) (**c**) (Original magX 25000). In striated duct showing loss of basal folding (red arrows), ruptured mitochondria (M), and swollen RER (R) (**d**) (Original magX 10000). In excretory duct showing clumping of chromatin and irregular nuclear membrane (N), swollen and ruptured mitochondria (M), dilated RER(R) and cytoplacmic vacuoles (V) (**e**) (Original magX 10000)

In the Drug group, many acinar cells exhibited diverse patterns of structural changes. Some nuclei displayed irregular outlines and chromatin clumping, while others exhibited noticeable shrinkage with condensed chromatin (Fig. 6a). In these cells, the RER showed varying degrees of damage, ranging from luminal dilatation to discontinuity, fragmentation, and loss of ribosomes (Fig. 6a, b). The secretory granules exhibited varying electron densities with ill-defined outlines and were predominantly less electron-dense than the Control group. Large cytoplasmic vacuoles and enlarged whorled mitochondria were also detected in the acinar cells (Fig. 6a). Mitochondria appeared with various forms of damage, such as dilatation with loss of cristae, and an enlarged whorled pattern (Fig. 6a, b, c). Moreover, ductal changes were also evident. Ultrastructural features of striated duct cells included varying degrees of mitochondrial damage, such as dilatation, loss of cristae, and even rupture; chromatin clumping; and an irregular nuclear membrane. Additionally, swollen RER with detached ribosomes, loss of basal folding, and a dilated basement membrane were observed (Fig. 6d). The excretory duct exhibited chromatin clumping, an irregular nuclear membrane, swollen and ruptured mitochondria, and dilated RER with loss of ribosomes (Fig. 6e).

**Fig. 7:**
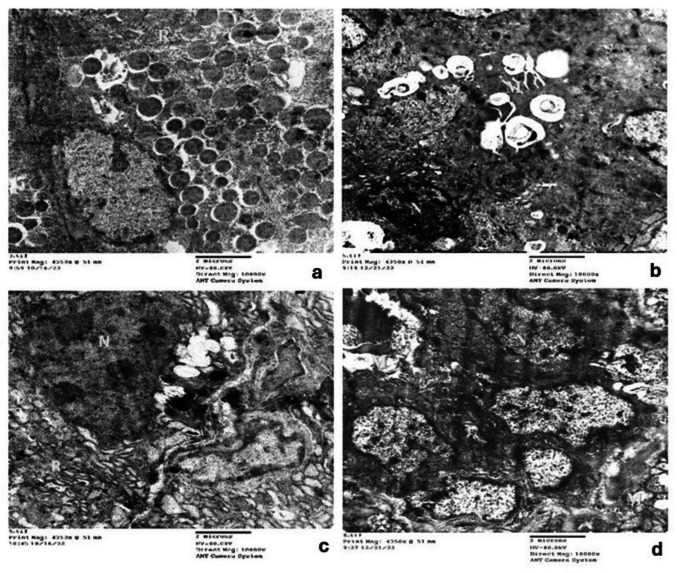
A photomicrograph of parotid salivary gland in Drug + MPE group showing: vacuolation in acini around zymogen granules (red arrows), and less dilated RER (R), intact desmosomes and basement membrane representing intercellular union (blue arrow heads) (**a**) (Original mag. X10000), Striated duct showing ruptured mitochondria (M) (**b**) (Original mag. × 10,000), nuclear membrane irregularities chromatin clumping (N), dilated RER with attached ribosomes (R) and dilated basal folding (red arrows) (**c) (**Original mag.x 10,000). In execratory duct showing clumping of chromatin (N) and ruptured mitochondria (R) (**d**) (Original mag. × 10,000)

In the Drug + MPE group, ultrastructural examination of acinar cells revealed a notable degree of preservation. Most nuclei appeared open-faced with intact nuclear membranes. However, a few nuclei still exhibited membrane irregularities and some chromatin clumping. While some acinar cells maintained intact basement membranes and intercellular junctions, these features were compromised in other areas, accompanied by membrane irregularities. Additionally, areas of vacuolations around zymogen granules and less dilated RER with attached ribosomes were observed **(**Fig. 7a**)**. Furthermore, striated ducts demonstrated a lesser degree of mitochondrial rupture in some areas, along with reduced perinuclear vacuolation, fewer nuclear membrane irregularities, and less chromatin clumping. The RER in these cells also appeared less dilated with more attached ribosomes, and basal folding showed less dilation **(**Fig. 7b, c**)**. In the excretory duct, cells presented with fewer destructive features, showing reduced chromatin clumping and nuclear irregularities, as well as less dilated RER with attached ribosomes **(**Fig. 7d**)**. All these findings suggest a partial recovery in both acinar and ductal cells of the parotid glands.

## Discussion

Fruit and vegetable by-products, such as mango peel, represent a significant, yet often underutilized, source of bioactive compounds and potential health-promoting antioxidants (Duda-Chodak and Tarko 2007). The disposal of mango peel, a substantial byproduct of the mango processing industry, poses an environmental challenge. However, the peel itself is exceptionally rich in diverse phytochemicals, including mangiferin, polyphenols, anacardic acid, quercetin 3-O-galactoside, and vitamins (C, E, and carotenoids) (Castro-Vargas et al. 2019; Maldonado-Celis et al. 2019; Quintana et al. 2021). Thus, exploring the use of mango peel for its health benefits offers a promising avenue of research.

It’s commonly believed that female subjects introduce more variability into studies because of their estrous cycle, though recent research (Dayton et al. 2016) challenges this idea. In response to this evolving understanding, the National Institutes of Health has even implemented policies to increase the use of both male and female cells and organisms in studies. Nevertheless, evidence points to a potential impact of hormonal fluctuations on the salivary glands in female rats (Mohamed et al. 2015; Zhao et al. 2022).

The carboplatin/5-FU regimen frequently causes significant adverse events, often requiring dose modification or treatment discontinuation. Chemotherapy-induced oral mucositis is a common effect, increasing the risk of malnutrition and worsening patient outcomes. Moreover, salivary gland dysfunction and reduced saliva secretion compromise both oral mucosal health and hygiene (Fujiwara et al. 2022).

Consequently, this study was conducted to investigate the potential cytoprotective effects of MPE and its ability to mitigate the adverse effects of carboplatin/5-FU-induced parotid gland injury and hematopoietic toxicity in male albino rats.

Our findings revealed a significant reduction in body weight among carboplatin/5-FU-treated rats. Concomitant administration of MPE with carboplatin/5-FU resulted in a notable increase in body weight compared to the carboplatin/5-FU group. This observation may be attributed to the amelioration of parotid salivary gland injury.

Intriguingly, the carboplatin/5-FU rats in the Drug group exhibited a decrease in blood glucose levels, a finding that diverges from the anticipated chemotherapy-associated hyperglycemia often attributed to impaired insulin production (Guo et al. 2018). The observed hypoglycemia is likely a consequence of dysphagia and malnutrition stemming from chemotherapy-induced salivary gland dysfunction (Fujiwara et al. 2022; Oguz et al. 2020). This interpretation is corroborated by the significant weight loss noted in this group. However, the blood glucose-lowering effect of carboplatin/5-FU was not influenced by the co-administration of MPE. These findings align with those of Mistry et al., who reported significant body weight gain and reduced fasting glucose levels in animals treated with MPE for 21 days (Mistry et al. 2023). In addition, studies indicate the mango peel ethanol extract, rich in bioactive compounds, can effectively mitigate the progression of diabetes and its associated complications. (Gondi and Prasada Rao 2015).

Furthermore, the current study suggests that MPE possesses myeloprotective properties, as evidenced by the increase in total hematopoietic cell count observed in the group receiving concurrent MPE and carboplatin/5-FU treatment compared to the marked reduction seen in the carboplatin/5-FU group alone. The observed improvement in the tested hematological parameters in this group may be attributed to the antioxidant properties of MPE, including vitamins, minerals, and polyphenols, which can neutralize reactive oxygen species (ROS).

Studies have documented myelosuppression associated with the carboplatin/5-FU drug combination, as well as the potential myeloprotective effects of certain plant extracts or their active metabolites in chemotherapy. Specifically, Galot-Linaldi et al. (2021) reported that while the carboplatin/5-FU regimen causes myelosuppression, the active metabolite anacardic acid exerts a cytoprotective effect against the resulting hematological toxicity. (Galot-Linaldi et al. 2021).

In accordance with the results of the present study, it has been reported that gallic acid exhibits cytoprotective properties for hematopoietic cells (Nair and Nair 2013). Moreover, gallic acid exerts its anti-inflammatory effects primarily through the modulation of the Mitogen-Activated Protein Kinase (MAPK) signaling pathway. Furthermore, its mechanism of action includes significant anti-oxidative activities(Bai et al. 2021).

In addition, rutin also contributes substantially by actively mitigating the oxidative stress that initiates and perpetuates the inflammatory response (Muvhulawa et al. 2022).

The pathogenesis of salivary gland damage is a complex process involving oxidative stress, inflammation, and cell death (Elmansy and Hegazy 2020). Numerous chemotherapeutic agents can impair salivary gland function, and it is well established that chemotherapy-induced oxidative stress correlates with therapeutic efficacy. Studies show that ROS generation can lead to genomic instability in tumor cells, promoting cellular apoptosis, senescence, or autophagy (Zhang et al. 2018). Moreover, oxidative stress contributes to cell damage, in both tumor and normal cells, induced by platinum-based compounds (Numazawa et al. 2011).

The effects of chemotherapeutic drugs on the major salivary glands of albino rats have been documented in previous histopathological research (Bomfin et al. 2017; Yurdabakan et al. 2022). Degeneration, manifesting as vacuolization in acinar and ductal cells, nuclear pyknosis, and subsequent apoptosis, has been documented. The escalating severity of these pathological changes is likely a consequence of the drug’s toxic effects, particularly at higher chemotherapeutic doses (Yurdabakan et al. 2022). This comes in accordance with the current study, as the histological analysis of the parotid gland tissue in the Drug group revealed significant structural damage to both acinar and ductal cells, loss of basal striation in striated ducts, and loss of pseudostratification in excretory ducts. Additionally, stagnant secretions and inflammatory cell infiltration were observed. Bomfin et al. demonstrated that 5-FU treatment can lead to significant salivary gland damage, associated with inflammation and oxidative stress, causing adverse effects on salivary flow rate and composition (Bomfin et al. 2017).

The ultrastructural examination in the current study provided strong corroboration of microscopic findings, illustrating various abnormalities in acinar and ductal cellular morphology. These alterations encompassed irregular nuclei, chromatin clumping, nuclear pyknosis, endoplasmic reticulum damage, aberrant secretory granules, cytoplasmic vacuolization, mitochondrial damage, and irregular nuclear membrane. Additionally, the RER appeared swollen with detached ribosomes, loss of basal folding, and a dilated basement membrane. Importantly, MPE significantly attenuated many of the histological alterations induced by carboplatin/5-FU treatment. This improvement is likely due to MPE’s enhanced antioxidant properties, stemming from its rich polyphenolic content. MPE’s elevated antioxidant capacity effectively inhibits the generation of ROS, which may be responsible for this protective effect (Ajila et al. 2007). Based on these findings, it is reasonable to hypothesize that MPE may exhibit cytoprotective properties.

## Limitations

A significant limitation of this investigation is the lack of functional correlations to the observed structural preservation of the salivary glands. Specifically, critical functional readouts, such as stimulated salivary flow rate and detailed compositional analysis, were precluded by inherent methodological constraints in the current experimental setup. Future research is essential to overcome these limitations and establish a definitive link between the demonstrated morphological integrity and clinically relevant salivary gland function.

## Conclusion

This study concludes that MPE offers multifaceted protective benefits, including myeloprotection for hematopoietic cells, mitigation of weight loss, and reduction of elevated blood glucose in the context of carboplatin/5-FU treatment. Crucially, MPE co-administration significantly alleviated the carboplatin/5-FU-induced injury to the parotid gland. Future investigations are essential to fully characterize the mechanisms responsible for these effects and to establish optimal strategies for MPE’s therapeutic integration.

## Data Availability

The datasets used during the current study are available from the corresponding author on reasonable request.

## Abbreviations

MPE: Mango peel extract
5-FU: 5-Fluorouracil
TEM: Transmission electron microscopy
SPSS: Statistical Package for Social Sciences
SD: Standard deviation
RER: Rough endoplasmic reticulum
ROS: Reactive oxygen species
MAPK: Mitogen-Activated Protein Kinase
